# Pilli Kai Score: A Proposed Digital Twin Framework Integrating Radiomics and Biomarkers for Enhanced Lung Nodule Risk Stratification

**DOI:** 10.7759/cureus.104310

**Published:** 2026-02-26

**Authors:** Zain Khalpey, Suchitra Pilli

**Affiliations:** 1 Cardiothoracic Surgery Department, HonorHealth, Scottsdale, USA; 2 Heart and Lung Surgery Group, HonorHealth, Scottsdale, USA

**Keywords:** blood biomarkers, clinical utility, cost-effectiveness, digital twin, lung cancer risk, pet-ct, prediction model, pulmonary nodules, radiomics, risk stratification

## Abstract

Indeterminate pulmonary nodules are a frequent and challenging finding in both screening and incidental imaging. Existing clinical prediction models provide structured estimates of malignancy risk but remain limited in precision, particularly for patients with intermediate pre-test probability. This technical report proposes the Pilli Kai Score, a digital twin framework that integrates clinical variables, radiomic features, blood-based biomarkers, and positron emission tomography (PET) data into a unified probability estimate for malignancy.

The framework outlines a multi-modal modeling strategy incorporating validated clinical predictors, standardized radiomics, biomarkers evaluated in pulmonary nodule populations, and PET categories when available. Prespecified validation targets include strong calibration across risk strata, an area under the receiver operating characteristic (ROC) curve exceeding 0.85, and a high negative predictive value to safely defer invasive procedures in benign disease, with comparative evaluation against established clinical models. No patient-level data are analyzed; instead, illustrative figures present the proposed workflow and anticipated performance benchmarks. If validated in multi-center studies, this framework could improve diagnostic accuracy, reduce avoidable interventions, and enable more personalized lung cancer care pathways.

## Introduction

The incidental discovery of pulmonary nodules on computed tomography (CT) scans represents a growing clinical challenge, driven by the increased use of cross-sectional imaging and expanded lung cancer screening programs. Foundational clinical prediction models, including the Mayo Clinic model [1], evidence-based practice guidelines [2], and Fleischner Society recommendations [3], provided structured approaches to malignancy risk estimation but remain limited in precision, particularly for nodules with intermediate risk. More recent models, such as the Brock (PanCan) score and subsequent validation studies, have improved discrimination in screening populations while highlighting ongoing performance variability across clinical contexts [4-6].

The goal of this work is to define a new scoring framework that integrates clinical data, biomarkers, and radiomics into a single probability estimate. This paper introduces the conceptual framework for the Pilli Kai Score, a proposed risk stratification tool designed within a digital twin architecture. We hypothesize that integrating comprehensive clinical data, quantitative radiomic features extracted using standardized methods, and validated blood-based biomarkers can meaningfully improve malignancy prediction. This report outlines the proposed development pathway and validation strategy, positioning the Pilli Kai Score as a longitudinally adaptable, digital twin-based decision support tool.

## Technical report

Pulmonary nodule risk prediction has been extensively studied. Early clinical models established foundational predictors and remain widely used in practice [1]. Guideline frameworks subsequently refined evaluation strategies and management pathways for indeterminate nodules, emphasizing structured risk stratification and evidence-based follow-up [2,3]. Newer cohort-based models, including the Brock (PanCan) score, improved discrimination in screening populations, while external validation studies have underscored strengths and limitations across imaging conditions and patient populations [4-6].

Advances in artificial intelligence and radiomics have introduced quantitative imaging phenotypes that may provide complementary information beyond conventional clinical assessment [7,8]. This technical report describes the Pilli Kai Score, a proposed digital twin framework that integrates clinical predictors, quantitative CT radiomics, blood-based biomarkers, and optional positron emission tomography (PET) information to generate a patient-specific probability of malignancy for indeterminate pulmonary nodules.

Target population and intended use

The framework is intended for adults with indeterminate pulmonary nodules identified incidentally or through screening. Although no patient-level data are analyzed in this report, future validation efforts will focus on nodules measuring 8-30 mm with pre-test probabilities between approximately 5% and 65%, consistent with guideline-supported intermediate-risk decision-making contexts [1,2,4]. The score is envisioned as a clinical decision support tool when standard models or qualitative assessment leaves residual uncertainty.

Data requirements and harmonization

The proposed framework anticipates the standardized capture of clinical variables used in established models, including age, smoking history, nodule size, spiculation, upper-lobe location, and prior extrathoracic malignancy beyond five years, alongside quantitative CT radiomic features, validated blood-based biomarkers, and categorical PET information when available [1,4,9-12]. Radiomics extraction is intended to follow open and validated workflows with documented parameters to ensure reproducibility across scanners and institutions.

**Figure 1:**
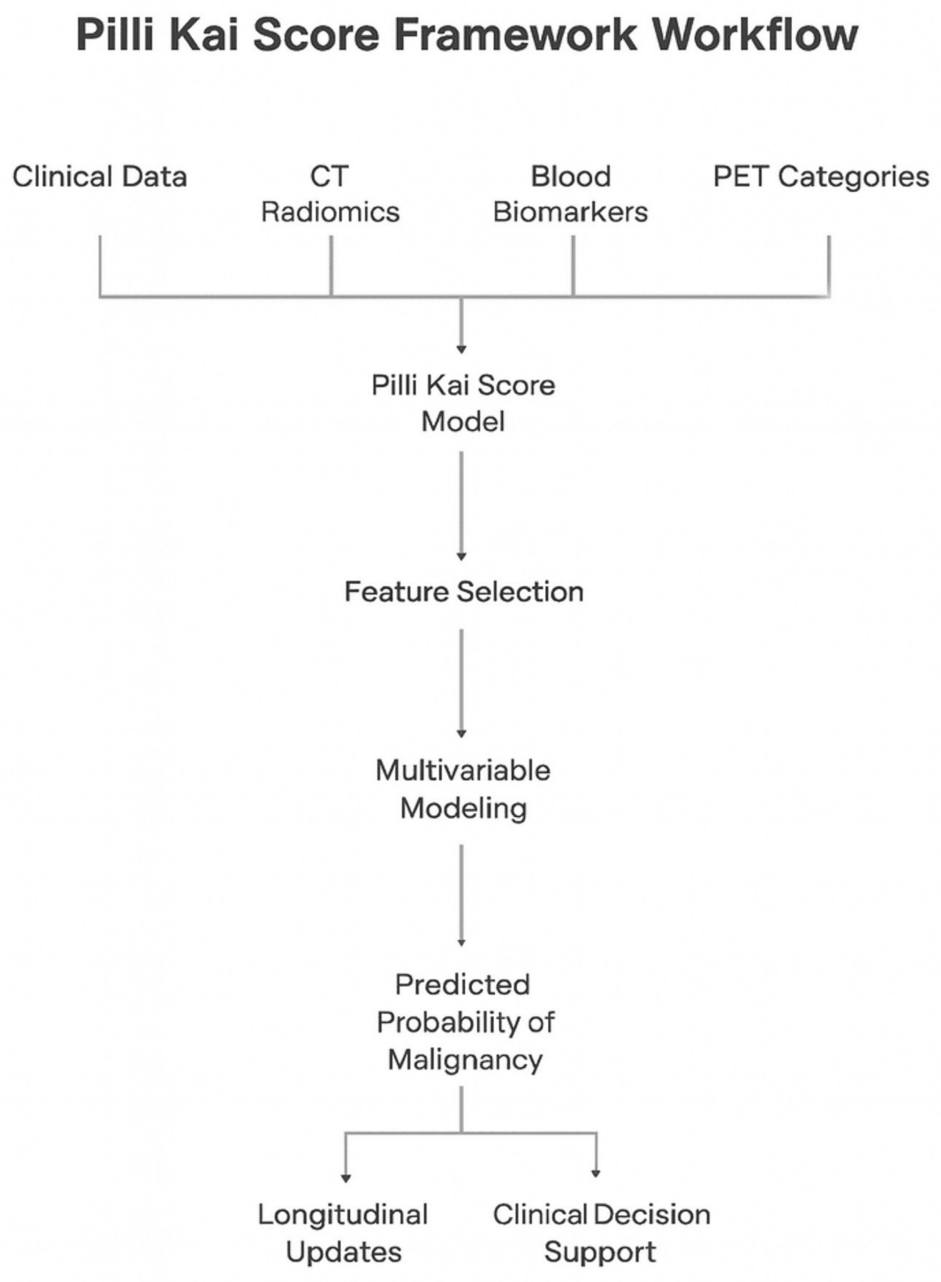
Conceptual Workflow of the Pilli Kai Score Framework Schematic representation of the proposed Pilli Kai Score framework. Clinical predictors (age, smoking history, nodule size, spiculation, location, and prior cancer history), quantitative CT radiomic features (extracted using PyRadiomics with IBSI-compliant protocols), blood-based biomarkers, and optional FDG PET categories are integrated into a digital twin model [7]. Feature selection (LASSO) and multivariable modeling generate a patient-specific probability of malignancy. The framework is designed to support longitudinal updates as new imaging or biomarker data become available, with the ultimate goal of guiding clinical decision-making and reducing unnecessary invasive procedures. Original figure created by Zain Khalpey CT, computed tomography; FDG, fluorodeoxyglucose; PET, positron emission tomography; LASSO, Least Absolute Shrinkage and Selection Operator; IBSI, Image Biomarker Standardization Initiative

The schematic depicts data ingestion and processing steps that integrate clinical variables, quantitative CT radiomics (preprocessing, segmentation, and feature extraction), blood-based biomarkers, and optional PET categories into a unified malignancy probability estimate via the Pilli Kai Score. Figure 1 emphasizes quality control, standardized radiomics per the Image Biomarker Standardization Initiative (IBSI), and provisions for future longitudinal updates within a digital twin architecture.

Image processing and radiomic feature extraction

Feature families should include first-order intensity statistics, shape, and texture matrices, bearing in mind prior studies that have cataloged radiologic signatures and underscored the need for robust external validation in screening and clinical cohorts [13-18].

PET information

When available, 18F-fluorodeoxyglucose (18F-FDG) PET uptake can be incorporated as an ordinal variable (none, faint, moderate, and intense) or via standardized uptake value categories, reflecting the established added value of PET in refining risk estimates in intermediate-probability nodules [17].

Image preprocessing should include voxel resampling and intensity normalization aligned with IBSI guidance, followed by segmentation that may be manual, semiautomated, or automated with expert verification, consistent with prior PET-augmented risk modeling approaches [19]. Radiomics extraction with PyRadiomics must preserve versioning, resampling grids, bin widths, and filtering parameters in a protocol log to enable replication across sites [20].

Evidence from large randomized lung cancer screening trials demonstrates that improved pulmonary nodule risk stratification has important downstream implications for lung cancer mortality and clinical outcomes [21].

Validation strategy and reporting

Transparent reporting will follow TRIPOD guidance, including the full specification of the final model, calibration assessments, decision-curve analyses, and clear descriptions of handling missing data and thresholds [22].

Publicly available datasets such as the Lung Image Database Consortium and Image Database Resource Initiative have supported standardized benchmarking and validation in pulmonary nodule research [23].

Illustrative performance targets and clinical use

**Figure 2:**
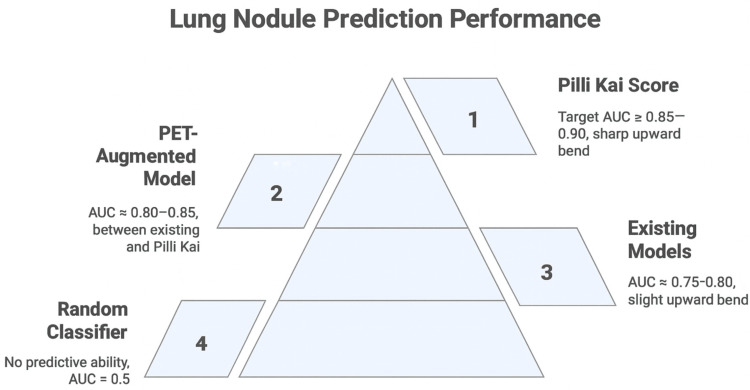
Conceptual Hierarchy of Lung Nodule Prediction Performance Conceptual pyramid depicting comparative performance of lung nodule malignancy prediction models. At the top, the proposed Pilli Kai Score represents the target benchmark (AUC ≥ 0.85-0.90) with a sharply improved predictive bend. Below this, PET-augmented models (AUC ≈ 0.80-0.85) provide intermediate performance, followed by existing clinical models such as Mayo or Brock (AUC ≈ 0.75-0.80). At the base, a random classifier illustrates no predictive ability (AUC = 0.5). This figure is illustrative and does not represent empirical results. Original figure created by Zain Khalpey AUC, area under the curve; PET, positron emission tomography

This technical report does not include patient-level results. For context, we predefine aspirational targets based on prior literature: an area under the receiver operating characteristic (ROC) curve exceeding 0.85-0.90 with strong calibration across risk strata, high negative predictive value to safely defer invasive procedures in benign disease, and improved classification performance over established comparators in external cohorts [4-12,14-19]. These targets are intended to guide future validation studies and should not be interpreted as achieved performance. This conceptual hierarchy of model performance is illustrated in Figure 2.

## Discussion

The Pilli Kai Score is proposed as a digital twin framework that integrates clinical, radiomic, biomarker, and PET data into a single malignancy probability estimate. Its rationale arises from known limitations of existing models, which may misclassify nodules in intermediate-risk scenarios despite prior validation [1,4-6]. By combining complementary data modalities, the framework aims to enhance discrimination, improve calibration, and enable longitudinal refinement of risk estimates.

Limitations and future directions

At present, the Pilli Kai Score remains conceptual. No patient-level data have been analyzed, and all performance metrics represent aspirational targets rather than observed results. Substantial work remains, including standardized radiomics pipelines, multi-center validation, and the assessment of implementation feasibility. Transparent reporting following TRIPOD guidance will be essential [22]. Prospective studies will be required to establish clinical utility and cost-effectiveness.

## Conclusions

The Pilli Kai Score is introduced as a conceptual multi-modal digital twin framework that brings together clinical variables, quantitative radiomics, validated blood-based biomarkers, and PET imaging into a single malignancy probability estimate for pulmonary nodules. The intent is to move beyond the limitations of existing clinical models and offer a tool that reflects the complex interplay of patient characteristics, imaging phenotype, and biologic signals. By embedding the framework within a digital twin architecture, the model has the theoretical capacity for longitudinal updating, adapting to new imaging or biomarker data as patients undergo surveillance.

At present, the Pilli Kai Score remains a proposal. The framework has not yet been validated on patient-level data, and its true performance, calibration, and clinical utility are unknown. The next steps will require rigorous development, standardized radiomics pipelines, and multi-center validation across diverse populations and imaging protocols. In addition, the careful evaluation of implementation feasibility, cost-effectiveness, and integration into clinical workflows will be necessary. If these challenges are successfully addressed, the Pilli Kai Score has the potential to reduce unnecessary invasive procedures for benign nodules, expedite the timely diagnosis of malignant disease, and ultimately contribute to more precise and personalized lung cancer care.
